# Effect of Ni Content on the Dissolution Behavior of Hot-Dip Tin-Coated Copper Wire and the Evolution of a Cu–Sn Intermetallic Compound Layer

**DOI:** 10.3390/ma18081714

**Published:** 2025-04-09

**Authors:** Qi Wang, Jinhan Zhang, Song Niu, Jinjin Fan, Shijun Tang, Shihong Tang, Ningkang Yin, Jingxuan Liu, Mingmao Li

**Affiliations:** 1 Faculty of Materials Metallurgy and Chemistry, Jiangxi University of Science and Technology, Ganzhou 341000, Chinazhangjhsch@163.com (J.Z.);; 2 Anhui Xinke New Materials Co., Ltd., Wuhu 241000, China; 3 Jiangxi Jinyi Nonferrous Metals Co., Ltd., Yingtan 335000, China

**Keywords:** Cu–Sn interface, hot-dip tin plating, intermetallic compounds, dissolution

## Abstract

The traditional hot-dip tinning processes face challenges in controlling excessive copper dissolution and interfacial instability. This study involved designing a dissolution experiment using the hot-dip tin plating process. Through microscopic characterization and dissolution kinetics analysis, it systematically revealed the regulatory mechanism of trace Ni addition (0–0.5 wt.%) on the dissolution behavior and interfacial reaction of copper wire in a tin alloy melt. The experiment showed that Ni atoms formed a (Cu_1−x_,Ni_x_)_6_Sn_5_ ternary phase by replacing Cu in the Cu_6_Sn_5_ lattice, resulting in a transformation of the grain morphology of the IMC layer from equiaxed to fibrous. At the same time, the addition of Ni changed the kinetics of the interfacial reaction, effectively increasing the activation energy from 40.84 kJ/mol in the pure Sn system to 54.21 kJ/mol in the Sn-0.5Ni system, which extended the complete dissolution time of the copper wire at 573 K by three times.

## 1. Introduction

Tin-coated copper materials have gained extensive applications in electronic information technology, telecommunications, and aerospace industries due to their exceptional corrosion resistance, workability, and electrical conductivity [1]. In recent years, their expanding utilization in connector fields has significantly enhanced the importance of tin-coated copper strips. With increasingly stringent requirements from new energy sectors and aerospace equipment for materials capable of stable operation under complex conditions involving high temperatures and humidity [2,3,4], higher demands have been imposed on both the surface quality of tin coatings and the operational reliability of tin-coated copper strips. The findings of this study specifically address the critical need for high-temperature resistant interconnects in aerospace power modules and precision microelectronic packaging, where controlling copper dissolution and stabilizing interfacial reactions are paramount for long-term reliability.

Traditional tin plating processes for copper strips primarily include electroplating and electroless plating. In electroplating, copper strips are immersed in tin salt solution where an electrical current drives the reduction of tin ions to metallic tin, forming the coating [5,6,7]. While electroplating is recognized for producing uniform coatings with excellent corrosion resistance, solderability, and high surface finish, it suffers from high energy consumption, severe environmental pollution, and substantial equipment costs [8]. Electroless plating involves immersing copper strips in chemical solutions rich in tin ions, utilizing surface chemical reactions to reduce tin ions into metallic coatings [9,10,11]. Although both electroplating and electroless plating can create elemental tin layers on copper substrates, their environmental impacts, low production efficiency, and inadequate performance in meeting requirements for miniaturized high-precision integrated circuit packaging and stringent bonding strength make them increasingly incompatible with modern energy conservation and emission-reduction standards. Hot-dip tinning has emerged as a novel continuous coating technology, where copper strips undergo sequential immersions in molten tin baths. Yin et al. [12] optimized the hot-dipped tinning process (260–280 °C, 6–10 s) to fabricate uniform Sn coatings with Cu_6_Sn_5_ IMC layers, ensuring enhanced electrical conductivity, corrosion resistance, and solder-joint reliability. This methodology integrated microhardness monitoring, SEM-EDS interfacial analysis, and standardized pretreatment procedures (alkaline cleaning followed by flux pretreatment) to guarantee coating uniformity and process stability throughout the manufacturing cycle. This process facilitates the formation of intermetallic compound (IMC) layers between copper substrates and tin, creating more stable and strongly bonded coating structures [13,14], thus presenting an effective solution to these challenges.

The dissolution behavior of copper substrates during tin plating involves three stages: dissolution (wetting of the solid–liquid interface and atomic interdiffusion), diffusion (migration of solute atoms driven by temperature/concentration gradients), and reaction (formation of Cu–Sn IMC barrier layers). Current research on metal substrate dissolution in liquid metals has established various models [1,15,16]; Oliveira et al. [17] combined solidification experiments and mathematical modeling to analyze interfacial thermal conductance in Sn-0.5Al alloy systems with Cu/Ni substrates. Their results demonstrated that Al-rich IMCs formed at the substrate interfaces, with significantly more dendritic/cellular structures developing on Ni substrates compared to Cu during solidification. Consistently demonstrating that copper dissolution in molten tin directly correlates with temperature and duration-dissolution rate and increases with temperature while the total dissolution amount grows with prolonged exposure [18,19,20]. The rapid interfacial reaction between copper and pure tin during hot-dip processes leads to excessive copper consumption. Given nickel’s low solubility in Sn-based solders and slow IMC formation kinetics, nickel has become the preferred barrier material in industrial applications. Zeng et al. [21] utilized μ-XRF to investigate Ni segregation in (Cu_1−x_,Ni_x_)_6_Sn_5_ IMC layers at Sn-0.7Cu-0.05Ni/CuBGA interfaces, demonstrating effective suppression of Cu_3_Sn growth. Wang et al. [22] experimentally confirmed that Ni addition to Sn2.5Ag0.8Cu solder inhibits copper diffusion at Cu–Sn interfaces, reducing Kirkendall void density and enhancing interfacial reliability. S.L. Tay et al. [23] reported that incorporating Ni nanoparticles into Sn-3.8Ag-0.7Cu solder transformed interfacial IMC morphology from scalloped to planar structures, promoting (Cu_1−x_,Ni_x_)_6_Sn_5_ growth while suppressing Cu_3_Sn formation.

While existing studies have verified nickel’s inhibitory effects on copper dissolution and IMC regulation in Cu–Sn interfacial reactions, significant research gaps remain. Current investigations predominantly focus on nickel’s modification effects in multi-component solder systems (Sn-Ag, Sn-Cu, Sn-Pb), with limited exploration of Sn-Ni binary systems and associated interfacial reactions. Particularly, systematic studies are lacking regarding the quantitative analysis of nickel content (especially in the 0.1–0.5 wt.% micro-doping range) on copper dissolution kinetics and IMC growth patterns during tin plating processes. This study establishes a Sn-xNi (x = 0–0.5 wt.%) alloy system to systematically quantify the influence of nickel content on copper wire dissolution rate, IMC growth kinetics, and interfacial reaction activation energy. The developed quantitative model correlating Ni content with dissolution inhibition efficiency provides theoretical guidance for copper-wire corrosion protection technologies.

## 2. Materials and Methods

### 2.1. Raw Material Preparation

The study utilized commercial-grade pure tin (99.95 wt.%) as the base material. Sn-xNi alloys with target compositions (x = 0.1, 0.3, 0.5 wt.%) were prepared using a Sn-Ni master alloy (4 wt.% Ni). After precise weighing, all raw materials were melted in graphite crucibles under argon atmosphere protection. The alloying process involved heating to 400 °C with 30 min isothermal holding to ensure compositional homogeneity, followed by casting into alloy ingots. The chemical compositions of prepared alloys are listed in Table 1. Commercially available oxygen-free copper wire (99.9% purity, Ø2 mm) served as the substrate material. Pretreatment involved sequential degreasing in 5% NaOH solution, ultrasonic cleaning in absolute ethanol, and thorough drying. Prior to immersion testing, the copper substrates were flux-treated with 4% HBr solution to enhance surface activation.

### 2.2. Experimental Procedure

For each test, 200 g alloy ingots were melted in graphite crucibles using indirect heating through a liquid tin bath. The melt temperature was precisely controlled at target values (523 K or 573 K, ±5 K tolerance). Preprocessed copper wires were sectioned into 100 mm lengths and vertically immersed in the molten Sn-Ni alloy bath. The dissolution process was monitored through periodic diameter measurements: initial 5 min observation with 30 s intervals followed by extended 5 min intervals until complete dissolution. Each parameter set was tested in triplicate for statistical reliability.

### 2.3. Specimen Preparation and Characterization

Cross-sectional specimens of the Sn/Cu interface reaction zone were prepared through sequential grinding (SiC papers #800–#5000) and final polishing with diamond suspension. For interfacial morphology analysis, specimens at different dissolution stages were subjected to selective etching using 40% nitric acid–alcohol solution to expose the intermetallic compound (IMC) layer [24,25]. Microstructural characterization was performed using a field emission scanning electron microscope (FE-SEM, Mira3 LMH, TESCAN, Brno, Czech Republic) equipped with EDS. TEM specimen preparation involved mechanical thinning to 100 μm followed by precision thinning using focused ion beam (FIB) milling at the wire cross-section. The resulting electron-transparent lamellae were analyzed by transmission electron microscopy (TEM) for crystallographic structure determination and elemental mapping.

## 3. Results

### 3.1. Phase Composition and Microstructure of IMC Layer

**Figure 1:**
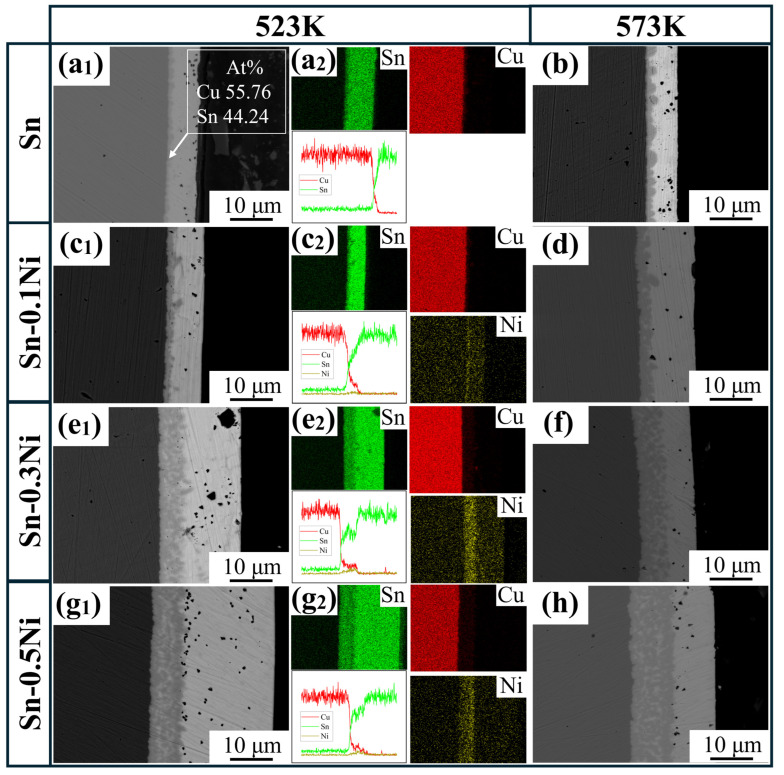
Interface microstructure and element distribution of copper wire in Sn-xNi (x = 0, 0.1, 0.3, 0.5) alloy melt: (**a_1_**,**c_1_**,**e_1_**,**g_1_**) 523 K; (**a_2_**,**c_2_**,**e_2_**,**g_2_**) EDS analysis corresponding to (**a_1_**,**c_1_**,**e_1_**,**g_1_**); (**b**,**d**,**f**,**h**) 573 K.

Figure 1 systematically investigates the interfacial reaction characteristics of copper wires dissolved for 10 s in Sn-xNi alloy systems (x = 0.1, 0.3, 0.5) and pure Sn melt at 523 K and 573 K. SEM morphological observations reveal distinct intermetallic compound (IMC) layers formed between the copper substrate (left) and tin coating (right). Figure 1(a_2_,c_2_,e_2_,g_2_) demonstrates the gradient distribution of Cu and Sn elements across the interface through EDS elemental mapping and line-scan profiles. The elemental mapping reveals significant Ni enrichment within the IMC layer. Simultaneously, line-scan profiles exhibit distinct Ni concentration peaks precisely aligned with the IMC region, demonstrating Ni’s tendency to accumulate at the Cu_6_Sn_5_ interface. The IMC layer is identified as Cu_6_Sn_5_ based on point-scan quantitative analysis (Cu: 55.76 at%, Sn: 44.24 at%) and phase diagram calculations, with atomic ratios closely matching the theoretical stoichiometry [26,27].

Temperature significantly modulates IMC layer growth. In pure Sn systems, elevating the temperature from 523 K to 573 K results in an increase in the IMC thickness from approximately 1 μm to around 3 μm, confirming that elevated temperatures accelerate Cu/Sn interdiffusion and promote Cu_6_Sn_5_ formation. Notably, Sn-Ni alloys also exhibit temperature sensitivity, with thicker IMC layers observed at 573 K compared to 523 K for all compositions. However, the thickness growth rate in Sn-Ni systems is lower than in pure Sn, suggesting that Ni addition mitigates temperature-driven interfacial reactions. Further analysis reveals dual effects of Ni on IMC evolution: (1) Sn-Ni alloy particles embedded within the IMC layer create unique morphological features distinct from pure Sn systems, potentially altering grain growth patterns; (2) increasing Ni content from 0.1 wt.% to 0.5 wt.% progressively enhances IMC thickness, likely due to Ni modifying interfacial reaction kinetics as a ternary component.

**Figure 2:**
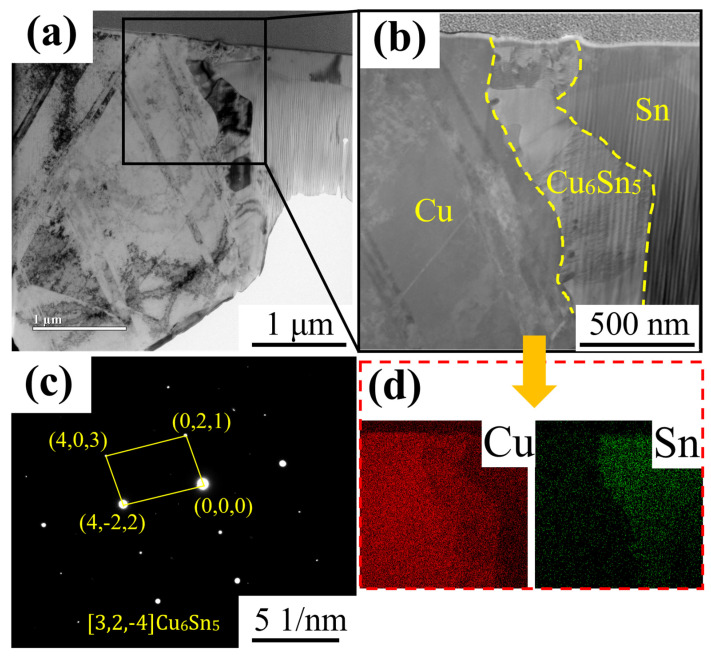
TEM images of copper wire dissolved in pure tin melt at 523 K for 10 s: (**a**,**b**) TEM images; (**c**) SAED pattern; (**d**) EDS-mapping.

To further confirm the phase composition of the IMC layer, this study employed TEM for multiscale characterization of copper wires dissolved in pure tin melt at 523 K for 10 s. Figure 2a shows the overall morphology of a thinned sample prepared via FIB, revealing a typical layered structure at the interface. High-resolution TEM imaging (Figure 2b) distinctly illustrates the layered configuration of the Cu substrate/IMC layer/Sn coating, where the η-Cu_6_Sn_5_ phase exhibits uniform thickness and continuous interfacial characteristics. Crystallographic analysis of the interface region using selected-area electron diffraction (SAED) confirmed the η-Cu_6_Sn_5_ phase. This structural consistency aligns with previous reports on η-Cu_6_Sn_5_ formation in Cu–Sn systems [28]. The diffraction pattern in Figure 2c, acquired along the [3,2,−4] zone axis, matches the superlattice reflection mode of η-Cu6Sn5. Indexed characteristic planes—including (0,2,1), (4,0,3), and (4,−2,2)—provide crystallographic confirmation of the IMC layer composition. The observed [3,2,−4] zone axis orientation differs from the orientation relationships reported in prior studies [28], indicating distinct nucleation mechanisms operative at liquid–solid interfacial reactions.

**Figure 3:**
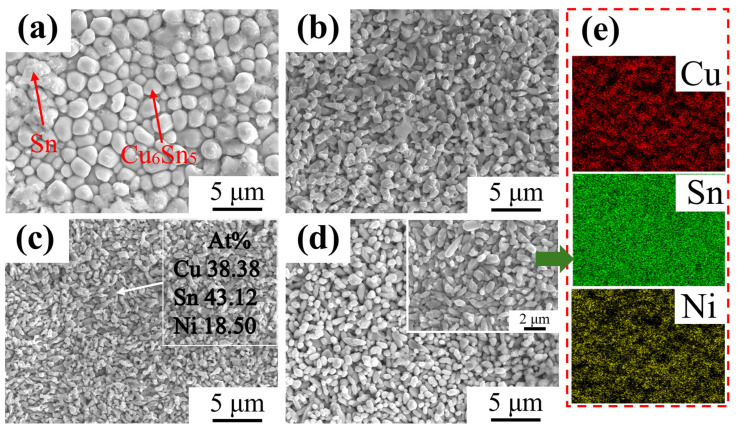
SEM images of IMC layers of copper materials dissolved for 10 s in different Sn-xNi alloy systems at 523 K: (**a**–**d**) Sn-xNi alloys (x = 0, 0.1, 0.3, 0.5); (**e**) EDS-mapping of (**d**).

Figure 3 systematically compares the morphology and EDS maps of IMC layers in copper wires dissolved for 10 s at 523 K in Sn-xNi (x = 0, 0.1, 0.3, 0.5) melts. The granular features correspond to Cu_6_Sn_5_ grains, while the fibrous residues on grain surfaces represent incompletely etched tin. In pure Sn melt (Figure 3a), Cu_6_Sn_5_ grains exhibit regular, rounded morphologies with relatively uniform sizes, suggesting that grain growth in Ni-free systems is governed by chemical reactions and diffusion mechanisms between Sn and Cu. Ni addition significantly alters IMC grain morphology (Figure 3b–d): grains become irregularly shaped, size distribution broadens, and refinement intensifies with increasing Ni content. The addition of Ni induces significant grain refinement of Cu_6_Sn_5_, which aligns with the findings reported by M.A.A. Mohd Salleh et al. [29]. EDS point analysis of a representative grain in Figure 3c reveals 38.38 at% Cu, 43.12 at% Sn, and 18.50 at% Ni. This indicates that Ni atoms partially substitute Cu in Cu_6_Sn_5_, forming (Cu_1−x_Ni_x_)_6_Sn_5_ solid solutions [20,30].

### 3.2. Microstructure Evolution of IMC Layer

**Figure 4:**
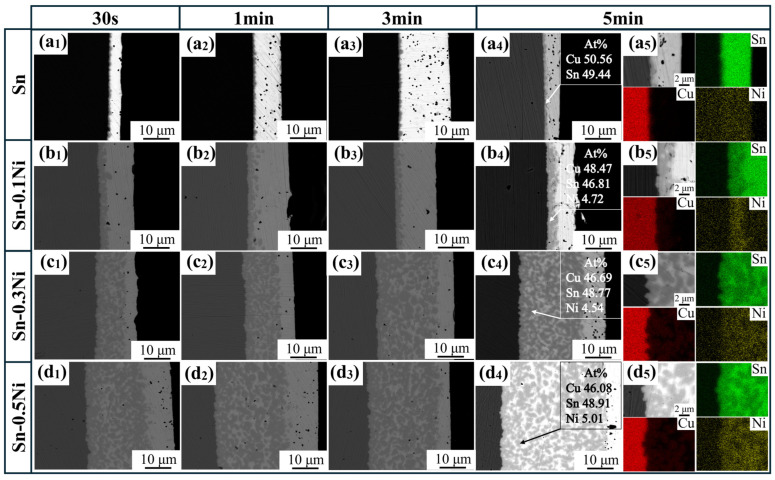
SEM images of sample cross-sections of copper material dissolved at different times in different Sn-xNi alloy systems at 523 K: (**a_1_**–**a_4_**) Sn; (**b_1_**–**b_4_**) Sn-0.1Ni; (**c_1_**–**c_4_**) Sn-0.3Ni; (**d_1_**–**d_4_**) Sn-0.5Ni; (**a_5_**–**d_5_**) EDS-mapping.

To observe the evolution of the intermetallic compound (IMC) layer at the Cu–Sn interface during isothermal dissolution at 523 K, continued isothermal dissolution tests were conducted on samples subjected to varying dissolution times (30 s, 1 min, 3 min, and 5 min). Figure 4 presents comparative SEM and EDS elemental mapping images of the IMC layer at the Cu–Sn interface in Sn-xNi (x = 0.1, 0.3, 0.5) alloy melts under different dissolution durations. In the pure Sn system (Figure 4(a_1_–a_5_)), the IMC layer maintained a constant thickness of approximately 1 μm throughout the dissolution process, showing no dependence on dissolution time. With Ni addition, elemental mapping analysis revealed that Ni preferentially enriches within the IMC layer, where the atomic ratios of Cu, Ni, and Sn align with the (Cu_1−x_Ni_x_)_6_Sn_5_ phase. The IMC layer thickness increased proportionally with Ni content. Furthermore, higher Ni concentrations amplified the time-dependent thickening of the IMC layer. This phenomenon arises from two mechanisms: (1) Ni acts as a surfactant, reducing the interfacial energy of Cu_6_Sn_5_ to promote nucleation; (2) Ni segregation in (Cu_1−x_Ni_x_)_6_Sn_5_ solid solutions impedes Sn diffusion, leading to grain refinement and increased grain boundary density.

**Figure 5:**
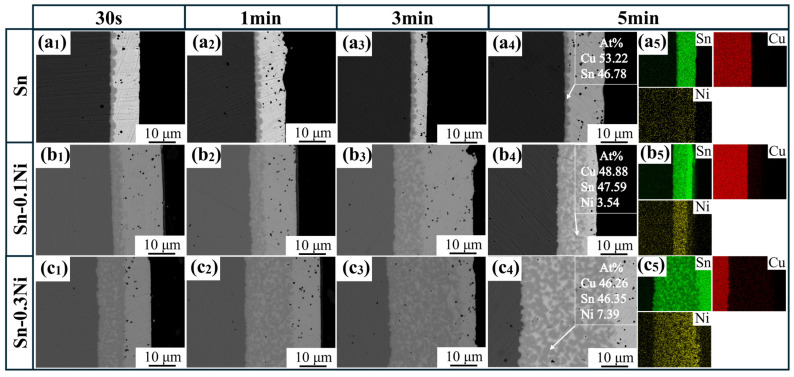
SEM images of sample cross-sections of copper material dissolved at different times in different Sn-xNi alloy systems at 573 K: (**a_1_**–**a_4_**) Sn; (**b_1_**–**b_4_**) Sn-0.1Ni; (**c_1_**–**c_4_**) Sn-0.3Ni; (**a_5_**–**c_5_**) EDS-mapping.

Figure 5 presents dissolution tests for Sn-xNi (x = 0, 0.1, 0.3) alloy systems at 573 K with the same time intervals (30 s, 1 min, 3 min, 5 min). Consistent with the trend observed at 523 K, Ni addition increases IMC thickness proportionally to its concentration. Additionally, IMC layers thicken with prolonged dissolution time. At identical Ni content and dissolution durations, IMC layers formed at 573 K are significantly thicker than those at 523 K, confirming that elevated temperatures accelerate IMC growth rates, enabling thicker layers within the same timeframe.

**Figure 6:**
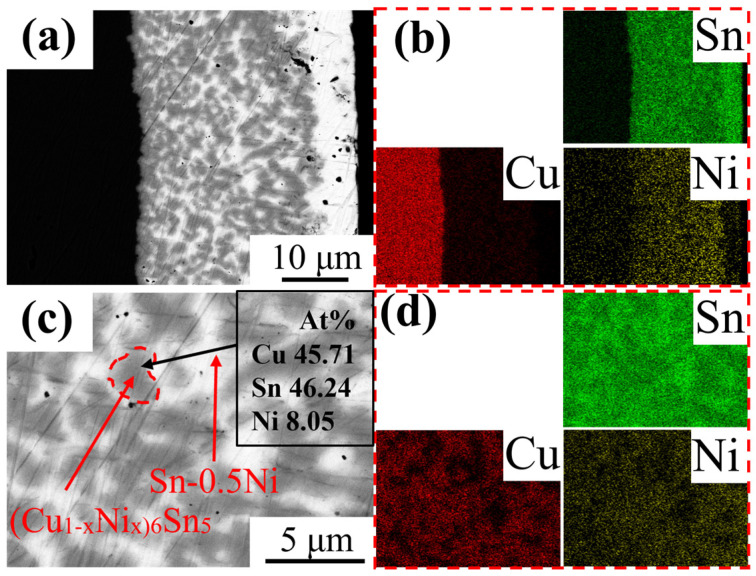
SEM and EDS images of copper wire material dissolved in Sn-0.5Ni at 573 K for 1 min: (**a**,**c**) SEM images; (**b**) EDS-mapping of (**a**); (**d**) EDS-mapping of (**b**).

Figure 6 displays the microstructure of the Sn-0.5Ni system after a 1 min etching treatment at 573 K. A distinct intermetallic compound (IMC) layer formed on the sample surface (Figure 6a). The corresponding EDS elemental mapping (Figure 6b) revealed significantly higher Ni concentration in the IMC layer compared to other regions, confirming selective segregation of Ni during interfacial reactions and its participation in the chemical reactions and structural formation of the IMC layer. Figure 6c shows an enlarged view of the IMC layer in Figure 6a, while Figure 6d presents the corresponding EDS elemental mapping. The co-localization of Cu and Ni enrichment areas demonstrates that Ni in the Sn alloy melt substitutes partial Cu in Cu_6_Sn_5_, forming (Cu_1−x_Ni_x_)_6_Sn_5_ compounds within the IMC layer.

**Figure 7:**
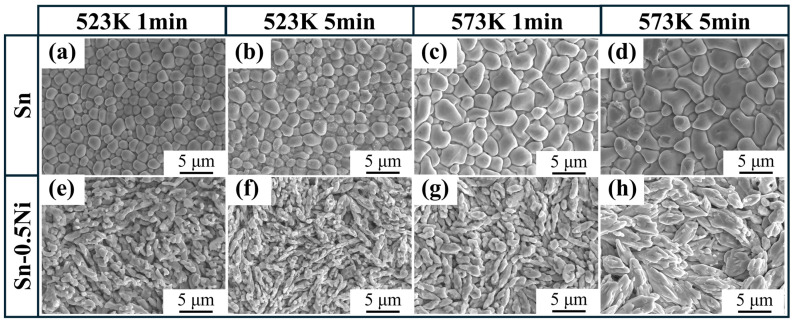
SEM images of IMC layer of copper wire after dissolution treatment at different temperatures (523 K/573 K) and times (1 min/5 min) in pure Sn and Sn-0.5Ni alloy melts: (**a**–**d**) Sn; (**e**–**h**) Sn-0.5Ni.

Figure 7 systematically characterizes the surface morphology evolution of IMC layers on copper wires after etching treatments in pure Sn and Sn-0.5Ni alloy melts under different temperatures (523 K/573 K) and durations (1 min/5 min). In the pure Sn system (Figure 7a–d), at 523 K with a 1 min treatment, the IMC layer consists of uniformly distributed near-spherical particles (2 μm diameter). Prolonged treatment to 5 min results in particle size broadening (2–5 μm) while maintaining clear grain boundaries. When temperature increases to 573 K, 1 min etching forms irregular polyhedral particles (3–8 μm), and 5 min treatment induces significant grain coarsening (5–10 μm) with boundary fusion, forming a dense continuous structure. The Sn-0.5Ni alloy system (Figure 7e–h) exhibits distinct morphological characteristics: at 523 K with 1 min treatment, the IMC layer shows aligned fibrous structures (aspect ratio 3:1). After 5 min etching, fiber length increases to 10 μm with enhanced orientation consistency. Under 573 K conditions, 1 min etching produces lath-shaped structures (3 μm width), which evolve into dense layered structures after 5 min treatment. Comparative analysis indicates that Ni addition transforms the initial IMC morphology from equiaxed grains to anisotropic structures, demonstrating stronger longitudinal growth tendency during subsequent treatments.

### 3.3. Dissolution Kinetics of Cu

To quantitatively analyze the effect of Ni addition on the dissolution rate of copper wires in molten Sn, this study recorded the complete dissolution time of copper wires in Sn melts under 523 K and 573 K conditions. The experimental results are summarized in Table 2.

The results show that in pure Sn melt, the complete dissolution time of copper wires at 573 K (300 °C) is significantly shorter than that at 523 K (250 °C). At 573 K, complete dissolution requires 20 min, while at 523 K, it takes 40 min, doubling the time. This indicates that lower temperatures significantly slow the dissolution rate of copper wires in molten Sn. When the temperature increases from 523 K to 573 K, the complete dissolution time of copper wires is drastically reduced, demonstrating that elevated temperature strongly promotes the dissolution process of copper wires in the pure Sn system. As the Ni content increases from 0 (pure Sn) to 0.1, 0.3, and 0.5 wt.%, the complete dissolution time of copper wires gradually increases. This confirms that Ni addition inhibits the dissolution of copper wires in molten Sn, with the inhibitory effect becoming more pronounced as the Ni concentration rises.

**Figure 8:**
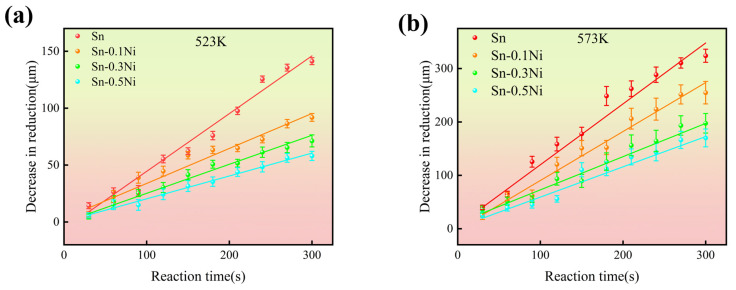
The curve of dissolution amount in Sn-xNi (x = 0, 0.1, 0.3, 0.5) alloy melt with respect to time for copper wire: (**a**) 523 K; (**b**) 573 K.

Figure 8 illustrates the variation of copper wire dissolution in tin alloy melts under different conditions as a function of dissolution time. In Figure 8a, with a dissolution temperature of 523 K, and in Figure 8b, with a dissolution temperature of 573 K, the horizontal axis represents reaction time while the vertical axis indicates the diameter reduction of copper wire to quantify dissolution amount. As the Ni content increases from 0 (pure Sn) to 0.1, 0.3, and 0.5, the dissolution rate of copper wire progressively decreases. This demonstrates that Ni addition inhibits the dissolution process of copper wire in tin alloy melts, with enhanced inhibitory effects at higher Ni concentrations. Notably, when temperature rises from 523 K to 573 K, the dissolution rates show significant acceleration, indicating the temperature’s substantial promoting effect on the dissolution process.

Based on the corrosion test data shown in Figure 8, the dissolution processes at both 523 K and 573 K follow the equation:

(1) $$X=X_{0}+kt^{n}$$

Herein, *X* represents the diameter reduction of the Cu substrate after dissolution, *X*_0_ denotes the diameter reduction before dissolution, *k* is the dissolution rate constant, *n* is the time exponent (both *k* and *n* being constants), and *t* is reaction time [31,32]. As shown in Figure 8, when *n* = 1, the dissolution curves demonstrate superior fitting with experimental data points under dissolution temperatures of 523 K and 573 K.

**Figure 9:**
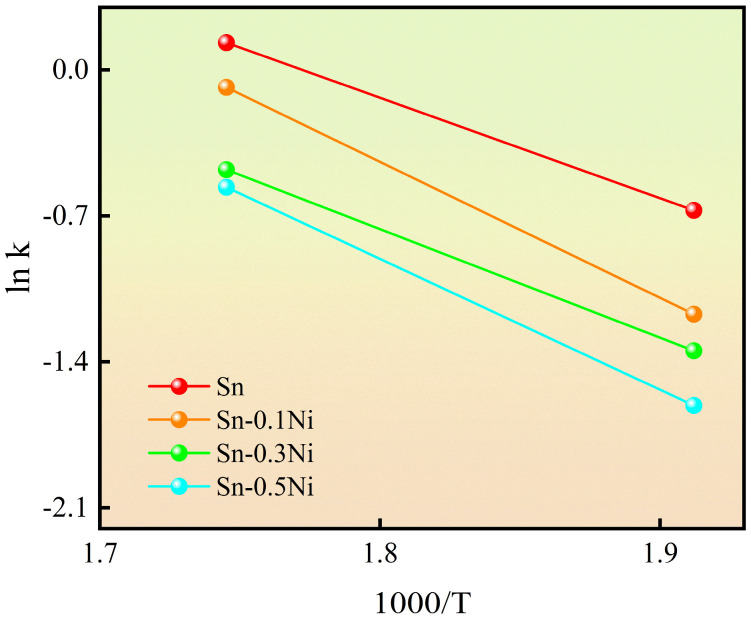
The temperature-dependent curve of the dissolution constant of copper wire in Sn-xNi (x = 0, 0.1, 0.3, 0.5).

Based on the dissolution rates shown in Figure 8, the dissolution rate constant *k* of copper wire under different temperatures and Ni addition levels was determined. The logarithmic Arrhenius plot of *k* versus time was constructed as illustrated in Figure 9.

The effective activation energy *Q* for copper dissolution under various conditions was further calculated using the Arrhenius equation:

(2) $$k=Aexp(-\frac{Q}{RT})$$

where *A* is the pre-exponential factor, *T* is the absolute temperature, *R* is the gas constant (8.314 J/(mol·K)), and *Q* represents the effective activation energy of the dissolution process [16,33]. The calculated *Q* values are summarized in Table 3.

For copper wire dissolution in pure Sn, the effective activation energy *Q* was determined to be 40.84 kJ/mol. With Ni addition, *Q* increased progressively with Ni content. Specifically, the effective activation energy *Q* reached 54.21 kJ/mol in Sn-0.5Ni alloy melt. This indicates that Ni addition effectively elevates the activation energy for Cu dissolution, thereby retarding the dissolution process. During experiments, continuous dissolution of Cu into the Sn melt alters the melt composition. However, the large volume of Sn melt employed in this study maintained an extremely low Cu concentration, effectively minimizing interference from dissolved Cu in the melt. Consequently, the measured dissolution quantities and calculated activation energies for Cu dissolution closely approximate ideal conditions.

## 4. Discussion

The measured activation energy of 40.84 kJ/mol for the pure Sn system in this study is lower than the previously reported values of 50.66 kJ/mol [34] and 51.91 kJ/mol [35]. This discrepancy primarily stems from experimental design characteristics: the substantial Sn melt quantity (200 g) maintained an ultra-low Cu concentration (<0.1 wt.%) at the dissolution interface, significantly weakening the concentration gradient driving force that dominates traditional parabolic models. As shown in Figure 8, the system exhibits linear dissolution kinetics (*n* ≈ 1), indicating interface reaction-controlled rather than diffusion-dominated mechanisms. According to solid–liquid interfacial reaction theory, parabolic law (*n* = 0.5) governs diffusion-controlled processes, while linear kinetics (*n* = 1) characterize reaction-controlled regimes. Although conventional wisdom suggests metal dissolution primarily follows diffusion control (*n* ≈ 0.5), Jeong et al. [36] observed anomalous *n* < 0.5 behavior under specific interfacial conditions like oxide layer formation. Notably, in this study, the density of Cu (~8.96 g/cm^3^) is approximately 1.3 times that of molten Sn (~6.99 g/cm^3^), causing gravitational sedimentation of dissolved Cu atoms from the upper interface to the Sn melt-bath bottom, thereby providing an additional driving force for Cu diffusion.

The elemental mapping results (Figure 1 and Figure 6) reveal a critical Ni redistribution mechanism during interfacial reactions. Ni atoms preferentially segregate into the IMC layer through substitutional replacement of Cu in the Cu_6_Sn_5_ lattice, forming (Cu_1−x_Ni_x_)_6_Sn_5_ ternary compounds. This substitution induces two significant microstructural modifications (Figure 3): (1) substantial grain refinement of the IMC layer increases grain boundary density and creates enhanced diffusion barriers, which aligns with the findings reported by M.A.A. Mohd Salleh et al. [29]; (2) grain morphology is transformed from equiaxed to anisotropic fibrous structures, as shown in Figure 7e–h. These microstructural evolutions directly contribute to the increased activation energy from 40.84 kJ/mol (pure Sn) to 54.21 kJ/mol (Sn-0.5Ni). Consequently, the complete dissolution time of copper wire at 573 K extended threefold from 20 min in pure Sn to 60 min in the Sn-0.5Ni alloy (Table 2), demonstrating nickel’s potent inhibitory effect on copper dissolution kinetics.

The dual mechanism of nickel’s action can be summarized as: (1) chemical modification through solid-solution strengthening of the IMC layer and (2) microstructural engineering of the interfacial barrier through grain refinement and morphological control. This comprehensive understanding provides fundamental guidance for developing high-performance tin-coated copper materials with tailored dissolution resistance for demanding applications in aerospace and microelectronic packaging.

## 5. Conclusions

This study systematically investigated the effects of Ni addition (0, 0.1 wt.%, 0.3 wt.% and 0.5 wt.%) in Sn melt on the dissolution kinetics of copper wire and the evolution of Cu–Sn IMC layers through hot-dip tinning experiments. The key findings are summarized as follows:

(1) Kinetic analysis revealed that Ni introduction significantly increased the effective activation energy (*Q*) of Cu dissolution (from 40.84 kJ/mol in pure Sn to 54.21 kJ/mol in Sn-0.5Ni), indicating that Ni inhibits the kinetic process of Cu atoms escaping the lattice by enhancing diffusion barriers. This mechanism extended the complete dissolution time of copper wire at 573 K from 20 min in pure Sn to 60 min in Sn-0.5Ni, with a threefold reduction in dissolution rate;
(2) In pure Sn systems, Cu_6_Sn_5_ grains at the interface exhibited regular equiaxed morphology with uniform size (~2 μm diameter at 523 K). With Ni addition, the IMC layer transformed into irregular fibrous grains with refined dimensions (aspect ratio of 3:1 in Sn-0.5Ni at 523 K). EDS elemental mapping confirmed that Ni atoms substitute Cu in the Cu_6_Sn_5_ lattice to form (Cu_1−x_Ni_x_)_6_Sn_5_ ternary phases, with Ni-rich regions overlapping Cu distribution. This demonstrates that Ni alters IMC growth patterns through solid-solution strengthening mechanisms;
(3) In pure Sn systems, Cu_6_Sn_5_ layer thickness was solely temperature-dependent (increasing from 1 μm at 523 K to 3 μm at 573 K) and independent of dissolution time, indicating diffusion-limited growth. In contrast, Ni-containing systems displayed time–temperature synergistic effects: at 573 K in Sn-0.5Ni, the IMC layer thickness increased from 3 μm (1 min) to 5 μm (5 min), with growth kinetics following an interface reaction-dominated mechanism (time exponent *n* ≈ 1), deviating significantly from the traditional diffusion-controlled model (*n* = 0.5, parabolic law). This discovery provides new theoretical insights for designing high-reliability tinned copper interfaces.

## Figures and Tables

**Table 1:** Chemical composition table of Sn-xNi Alloy (wt.%).

Alloy Designation	Sn	Ni
Sn	100	0
Sn-0.1Ni	99.9	0.1
Sn-0.3Ni	99.7	0.3
Sn-0.5Ni	99.5	0.5

**Table 2:** Time for complete dissolution of copper wire in Sn-xNi at 523 K and 573 K (min).

Temperature	Sn	Sn-0.1Ni	Sn-0.3Ni	Sn-0.5Ni
523 K	40	75	110	150
573 K	20	35	50	60

**Table 3:** Effective activation energy of copper wire in Sn-xNi (x = 0.1, 0.3, 0.5) (kj/mol).

Effective Activation Energy	Sn	Sn-0.1Ni	Sn-0.3Ni	Sn-0.5Ni
*Q*	40.84	43.67	52.14	54.21

## Data Availability

The original contributions presented in this study are included in the article. Further inquiries can be directed to the corresponding author.
